# Expression of autophagy and apoptosis-related factors in the periodontal tissue of experimental diabetic rats: a histomorphometric, microtomographic and immunohistochemical study

**DOI:** 10.7717/peerj.11577

**Published:** 2021-06-09

**Authors:** Youmin Mei, Xiang Shen, Xiaoqian Wang, Min Zhang, Qiao Li, Junyi Yan, Jiali Xu, Yan Xu

**Affiliations:** 1 Jiangsu Key Laboratory of Oral Diseases, Nanjing Medical University, Nanjing, China; 2 Department of Periodontology, Nantong Stomatological Hospital, The Affiliated Nantong Stomatological Hospital of Nantong University, Nantong, China; 3 Department of Stomatology, The Affiliated Hospital of Nantong University, Nantong, China; 4 Department of Periodontics, The Affiliated Stomatological Hospital of Nanjing Medical University, Nanjing, China

**Keywords:** LC3, Bax, Bcl-2, Diabetes, Periodontal tissue

## Abstract

**Objective:**

This study aimed to investigate the expression of autophagy-related factors microtubule-associated protein l light chain 3 (LC3) and the apoptosis-related factors BCL2-associated X protein (Bax) and B cell lymphoma-2 (Bcl-2) in the periodontal tissue of experimental diabetic rats. These data were used to explore the potential mechanism in diabetes-induced periodontal tissue lesions.

**Methods:**

A total of 32 Sprague Dawley (SD) rats were randomly assigned into diabetes (group D, *n* = 16) and control groups (group N, *n* = 16). The diabetic group was induced by intraperitoneal injection of 1% streptozotocin (STZ, 60 mg/kg) and the control group was injected with citrate buffer (0.1mol/L). Rats were sacrificed after 4 and 8 weeks of feeding and collected as D1, N1 groups and D2, N2 groups, and the maxilla were retained for analysis. The changes in periodontal tissue structure were observed by hematoxylin-eosin (HE) staining. The expression and distribution of LC3, Bax and Bcl-2 in the periodontium of the rats was detected by immunohistochemical (SP) staining.

**Results:**

Diabetic rats showed several changes compared to control animals including sparse alveolar bone trabecular structure, loss of the lamina dura and absorption of the local alveolar bone. The positive expression level of LC3 in the gingival epithelial, periodontal ligament and alveolar bone of group D1 was significantly higher than in the N1, N2 and D2 groups (*P* < 0.05). The level of Bax expression in the group D2 rats was significantly higher than those in the N1, N2 and D1 groups (*P* < 0.05), while the positive degree of Bcl-2 was significantly lower than those of other groups (*P* < 0.001). LC3 was negatively correlated with Bax and was irrelevant with Bcl-2; Bcl-2 was not correlated with Bax.

**Conclusions:**

The expression of LC3, Bax and Bcl-2 changes in the periodontal tissue of diabetic rats may indicate that autophagy and apoptotic are involved in the process of periodontal tissue damage in diabetic rats. These changes may be one of the mechanisms of periodontal tissue lesions.

## Introduction

The incidence of diabetes mellitus (DM) continues to grow at an increasing rate around the world. In China, the prevalence of DM ranks the highest in the world and the disease shows a younger trend (Wang, Li & Chen, 2018). DM describes a group of metabolic disorders that are characterized by persistent hyperglycemia resulting in many chronic complications (Paneni et al., 2013). People with diabetes have a three-fold elevated risk of periodontitis relative to those who do not have diabetes (Mealey & Ocampo, 2007). Periodontitis has been recognized as one of the important complications of diabetes (Bascones-Martinez, Gonzalez-Febles & Sanz-Esporrin, 2014). However, the exact mechanisms involved have not been elucidated fully. Several studies have indicated that apoptosis is an important form of cell death, described as a type of programmed cell death, plays a key role in the damage of periodontal tissues in diabetic periodontitis (Alikhani et al., 2005; Liu et al., 2006).

Autophagy is a critical energy recycling process. As a major regulated catabolic mechanism, autophagy is widely involved in the physiological and pathological processes of cells (Levine & Kroemer, 2008; Mizushima & Komatsu, 2011; Ott et al., 2016; Shintani & Klionsky, 2004). Autophagy is closely related to the occurrence of cell apoptosis (Maiuri et al., 2007; Mariño et al., 2014). In recent years, several studies have shown that autophagy and apoptosis are closely related to the occurrence and development of DM and its complications including diabetic nephropathy and diabetic cardiomyopathy (Hou et al., 2014; Kobayashi & Liang, 2015; Rosa et al., 2016; Shen et al., 2015). However, the relationship between autophagy and apoptosis is different under different diseases and conditions, and the relevant signal pathways regulated in cells were also different (Ding et al., 2010; Maiuri, Criollo & Kroemer, 2010; Xu et al., 2016b). However, this effect on periodontal tissues has been reported rarely.

Previous work from our laboratory showed that apoptosis is induced in advanced glycation end of products (AGEs)—treated human periodontal ligament cells (HPDLCs) (HPDLCs are the main cells present in periodontal connective tissue k) (Somerman et al., 1988). This was confirmed by the mitochondrial membrane potential depolarization, decreased Bcl-2 expression, increased Bax expression, and increased caspase-3 and PARP cleavage. Autophagy is also induced in AGEs-treated HPDLCs, as indicated by the conversion of LC3-II/LC3-I and the presence of autophagosomes. Whether it is related to the mechanism that diabetes mellitus influences periodontal destruction remains unclear (Mei et al., 2020).

In the present study, we investigated the expression levels of LC3 and apoptosis-related factors Bax and Bcl-2 in the periodontal tissue of experimental diabetic rats and explored their relationship and significance. Our findings provide a basis for the further study of periodontal tissue damage in patients with DM.

## Materials & methods

### Establishment of diabetic rat model and experimental grouping

A total of 36 male, clean-grade Sprague Dawley rats (6-week-old, weighing 185–225 g were obtained from the Laboratory Animal Center of Nantong University). Animals were fed with a standardized pelleted diet and provided water ad libitum. The laboratory ambient temperature was 18–22 °C, and the relative humidity is 50–60%. After one week of adaptive feeding, 32 rats were randomly divided into a DM group (group D, *n* = 16) and a normal control group (group N, *n* = 16). After fasting for 12 h, rats in group D were injected once intraperitoneally with 60 mg/kg body weight of 1% streptozotocin (STZ, Sigma–Aldrich, American) sterile solution to induce diabetes. Rats in group N were injected once intraperitoneally with an equal volume of citrate buffer. A total of 72 h later, tail vein blood was collected for blood glucose detection (Roche, Basel, Switzerland). Animals with consistent blood glucose levels ≥16.7 mmol/L for three consecutive days were regarded as successful diabetic model (Holzhausen et al., 2004). Blood glucose levels were measured at weekly intervals. If the blood glucose returned to normal or the rats died during the feeding process, the model was considered as a failure. A total of 14 rats were modeled and all of the 16 rats in the control group survived. Rats were maintained following the national standard set forth by the (GB 14925-2001). Approval of all animal protocols was obtained by the University Committee on Use and Care of Animals of Nantong University (S20200316-014, March 15, 2020).

### Specimen collection

A total of 4 weeks after successful modeling, 6 rats were randomly selected from groups D and N. Rats were sacrificed and collected as D1 and N1 groups. A total of 4 weeks later, 6 rats were randomly selected, sacrifice and recorded as the D2 and N2 groups. Rats in both groups were anesthetized by intraperitoneal injection with 10% chloral hydrate (3 ml/kg body weight). The maxilla and the mandible were scanned by micro-computed tomography (micro-CT, vivaCT80, Scanco, Switzerland). Rats were killed by rapid decapitation under an anesthetic overdose. The maxilla was rapidly isolated and the surrounding muscle tissue was removed. The maxilla was divided into two parts at the midline and immersed in a 4% paraformaldehyde fixative solution for histological examination.

### Micro-CT scanning

**Figure 1:**
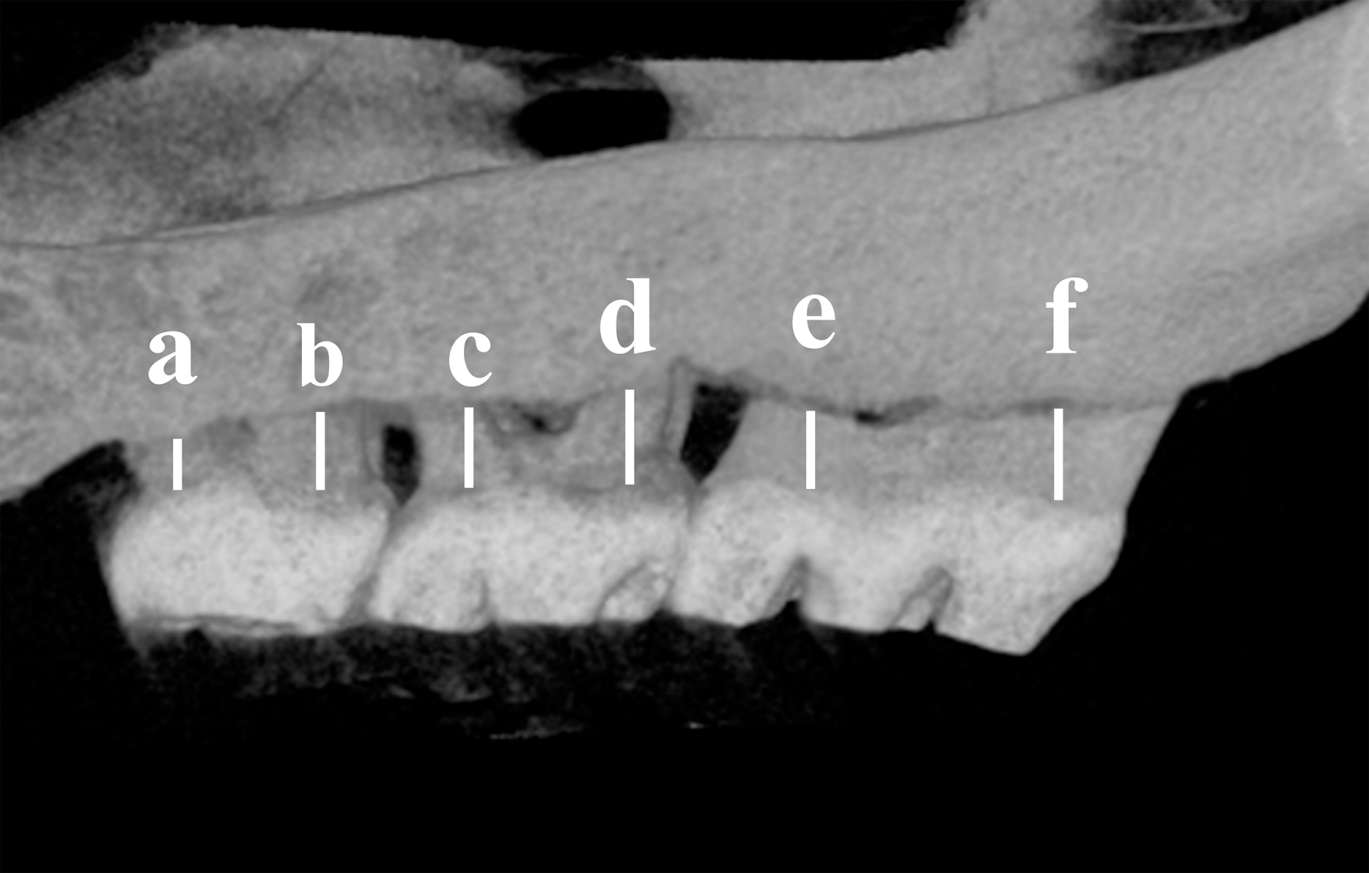
Schematic illustration of the distance measurements between the cement enamel junction (CEJ) and the alveolar bone crest (ABC). The distance from the CEJ to the ABC was measured at six points (a–f) indicated on the third molar (M3) to the first molar (M1).

To evaluate the alveolar bone loss, the maxillae were scanned by micro-CT at a 10 μm voxel resolution with an energy of 70 kV. The 3D reconstruction scans were used to measure the distance from the cementoenamel junction (CEJ) to the alveolar bone crest (ABC) on the buccal aspect of the maxillary molars (Pacios et al., 2015; Viniegra et al., 2018). The distance of alveolar bone loss (ABL) for each rat was measured at a total of 6 predetermined buccal sites from the CEJ to ABC of the three molars (illustrated in Fig. 1). The average of the measured values at the 2 sites was taken as the ABL value of each molar (illustrated in Fig. 1). The ABL value of each group was expressed as the mean ± standard deviation (mean ± SD) for all rats. All of the examinations were performed by two investigators who were blinded to the identification of the samples.

### Histological examination

The maxillae were fixed in a 4% paraformaldehyde solution at 4 °C for 24 h. The tissues were decalcified in 15% ethylenediaminetetraacetic acid (EDTA) for 12 weeks until pliable and then embedded in paraffin (Balci Yuce et al., 2016). The paraffin tissue blocks were cut into 5 µm thick sections for histological analysis.

#### Hematoxylin-eosin (HE) staining

Briefly, according to routine methods the paraffin-embedded sections were dewaxed and hydrated, and flushed with phosphate-buffered saline (PBS) for twice for 5 min. The sections were stained twice with hematoxylin for 5 min and washed with double distilled water for twice for 5 min. The samples were stained twice with eosin for 10 min and washed in double distilled water for twice for 5 min. Finally, after dehydration with gradient ethanol the slides were permeabilized with xylene, and mounted with neutral gum, and then observed under the microscope (two independent histopathologists evaluated the morphology of slides).

#### Immunohistochemistry (IHC) staining

According to conventional IHC staining protocol, after dewaxing and hydration, the paraffin sections were incubated with 3% H_2_O_2_ at room temperature for 20 min to inactivate endogenous peroxidase. Samples were washed with PBS and citrate for antigen repair. Sheep serum was used to block antigens. The sections were incubated with corresponding primary antibodies (anti-autophagy-related factors microtubule-associated protein l light chain 3 LC3, anti-apoptosis related factors BCL2-associated X protein Bax, and anti-B cell lymphoma-2, Bcl-2) at concentrations of 1:400 at 37 °C for 2 h and then overnight at 4 °C. All antibodies used for the experiment were obtained from Cell Signaling Technology (USA) and tested for the negative and positive control. Samples were incubated with a secondary antibody at 37 °C for 1 h and then DAB was used to develop the color at room temperature. The color development effect was observed under a microscope. Sections were restained with hematoxylin for 30 s and dehydrated through an ethanol gradient. The sections were treated xylene and mounted before being evaluated by two independent histopathologists.

All specimens were analyzed by light microscopy and recorded by two independent pathologists who were blinded to the specimen information. The semi-quantitative analysis of the IHC staining results was scored based on the degree of positive staining of most cells. The scores were graded on a 0 to 3 scale: 0 = negative staining, 1 = weakly positive staining, 2 = positive staining, 3 = strongly positive staining. The slices were placed under a high-powered microscope and 5 visual fields were randomly selected from each section for scoring (De Araújo et al., 2019; Loreto et al., 2014).

### Statistical analysis

The experimental data were analyzed and processed using Statistical Package for Social Sciences (SPSS) version 18.0. The normality test was performed on measurement data. Quantitative data were presented as mean ± Standard Error of Mean (SEM), and statistical analyses were performed using Students t-test and analysis of variance test. Ranked data were presented as median ± inter-quartile range (IQR), and Kruskal–Wallis test was performed. The correlation of variables was performed using Spearman correlation parameter. *P* < 0.05 was the significant difference.

## Results

### Construction of a diabetic rat model

In the diabetic group (group D1 and group D2), the blood glucose levels were significantly higher (*P* < 0.001), whereas the body weights were significantly less than that of control rats (group N1 and N2) (*P* < 0.05). This indicates that the diabetic rat model was successfully established (Tables 1 and 2).

**Table 1:** The body weights of the rats in both groups (g, mean ± SEM ).

Group	STZ administration			
	0 h	72 h	4 wk	8 wk
N (*N* = 16)	198.73 ± 18.12	234.45 ± 19.10	306.35 ± 16.21 (N1)	341.22 ± 23.71 (N2)
D (*N* = 14)	199.01 ± 17.25	201.78 ± 18.17*	208.25 ± 23.79 (D1)*	201.95 ± 30.51 (D2)*

**Notes:**

* *P* < 0.05 compared to the normal control group (group N).

Each value is expressed as the mean ± SEM.

**Table 2:** Blood glucose level of the rats in both groups (mmol/L, mean ± SEM).

Group	STZ administration			
	0 h	72 h	4 wk	8 wk
N (*N* = 13)	5.03 ± 0.89	5.48 ± 0.32	5.32 ± 0.76 (N1)	5.67 ± 0.56 (N2)
D (*N* = 15)	5.27 ± 0.54	24.39 ± 2.14^#^	26.78 ± 4.14 (D1)^#^	27.92 ± 4.08 (D2)^#^

**Notes:**

# *P* < 0.001 compared with the normal control group (group N).

Each value is expressed as the mean ± SEM.

### Radiographic assessment of alveolar bone loss

**Figure 2:**
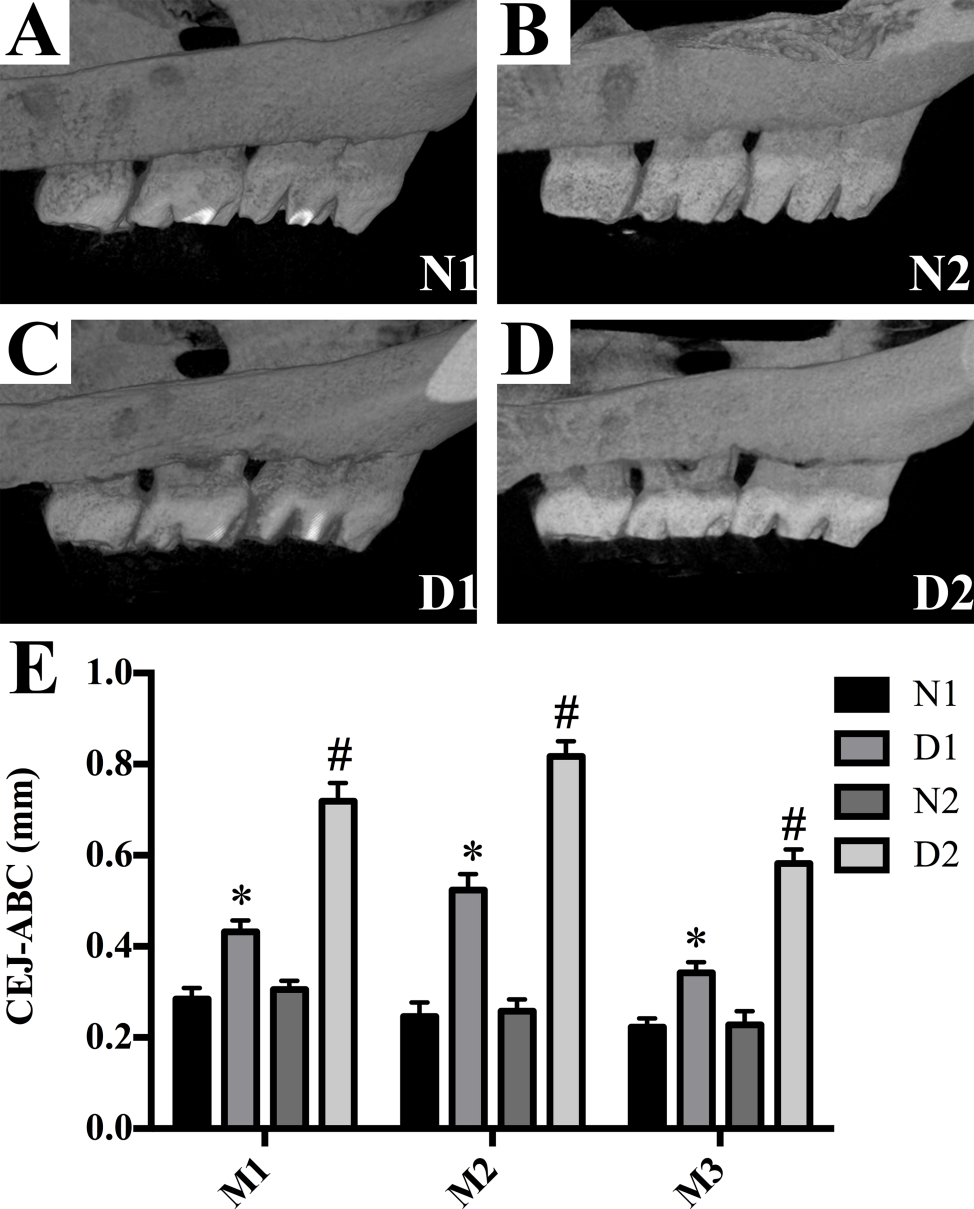
Alveolar bone loss was detected in M3 to M1 of each group. (A–D) Alveolar bone loss was detected in M3 to M1 of each group. (E) Data represent the mean values ± SEM, (*n* = 6 for each group). *p < 0.05 using ANOVA, compared to the normal control group (group N). #*p* < 0.001 using ANOVA, compared to the D1 group.

The diabetic rats (groups D1 and D2) showed a significant linear bone loss compared to the control animals (groups N1 and N2) (*P* < 0.05, Fig. 2). Compared with group D1, the ABL in group D2 was significantly increased (*P* < 0.001, Fig. 2).

### Histological analysis

Histological analysis of the area between the first and second molars in all groups revealed changes in the periodontal tissue structure of the rats by HE staining. In the N1 group, the gingival epithelium of the papilla between the molars was intact and there was no loss of connective tissue attachment to the surface of the tooth. The periodontal ligament fibers were orderly arranged and the height of the alveolar bone was normal.

In the D1 group, the gingival papilla was mostly normal, and the epithelial rete pegs proliferated to form a cord-like or mesh-like shape with attachment loss. The infiltration of inflammatory cells was found in gingival epithelium and lamina propria. Disordered arrangements of periodontal collagen fiber bundles and slight resorption of the alveolar bone were observed.

In the N2 group, the gingival papilla between the molars was mostly normal, the gingival epithelium was intact, the short epithelial rete pegs appeared in some of the gingival sulcus epithelium, and there was no attachment loss. The periodontal ligament fibers were regularly arranged, and the height of alveolar bone was normal.

In the D2 group, the partial loss of gingival papilla between molars and gingival epithelium erosion or ulceration was observed. The epithelial rete pegs proliferated and obvious attachment loss were observed. Massive infiltration of inflammatory cells was found in the gingival epithelium and the lamina propria. We also observed the irregular arrangement of periodontal collagen bundles, the dissolution and denaturation of some collagen fibers, significant alveolar bone absorption. IN summary, the periodontal tissues in group N1 and N2 were intact, and the gingiva (G), dentin (D), periodontal ligaments (PDL) and alveolar bone (AB) were observed.

**Figure 3:**
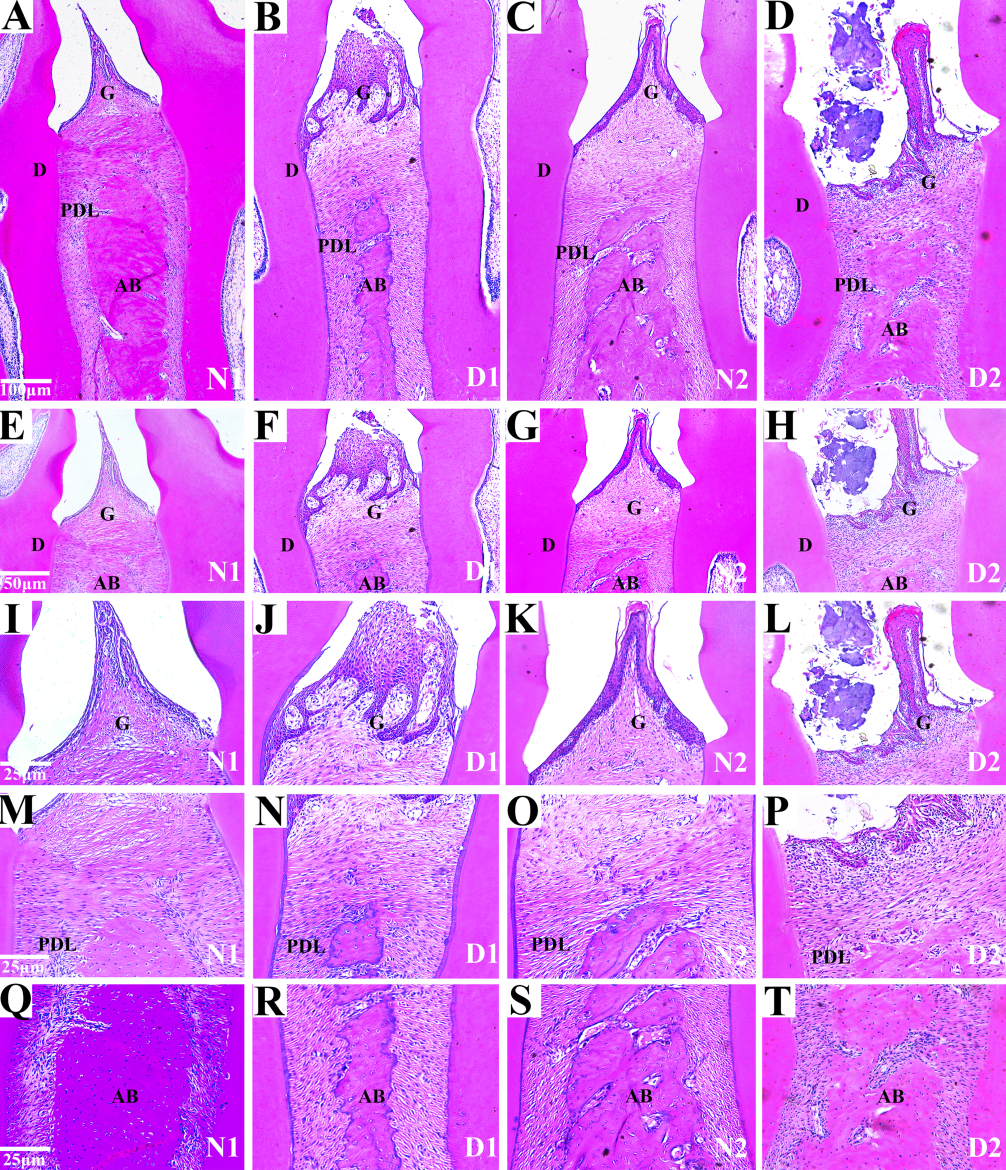
Histological analysis of the maxillae from rats with DM (D1 and D2) and control groups (N1 and N2). (A–H) Histological analysis of the maxillae from rats (N1 and N2) show the normal periodontium, the alveolar bone integrity and absence of or discrete cellular infiltration, and preserved alveolar bone. The periodontium from group D1 was mostly normal, the epithelial rete pegs proliferated. The infiltration of inflammatory cells was observed and the resorption of the alveolar bone was slight. The periodontium from DM rats (D2) showing alveolar bone resorption and accentuated inflammatory cell infiltration. Images are shown at 50×*g* and 100×*g* magnification. G (gingival); PDL (periodontal ligament); D (dentin); AB (alveolar bone); (I–T) Histological analysis of gingiva l (G), periodontal ligament (PDL) and alveolar bone (AB) respectively from rats with diabetes mellitus (D1 and D2) and control groups (N1 and N2). The images are shown at 200× magnification.

Compared to the group N1 and N2, the gingival epithelium erosion, inflammatory cell infiltration and the alveolar bone were absorbed locally in the rats in groups D1 and D2 indicating that DM caused periodontal inflammatory lesions or injuries in the rats (Fig. 3).

### Immunohistochemical analysis of LC3, Bax and Bcl-2

**Figure 4:**
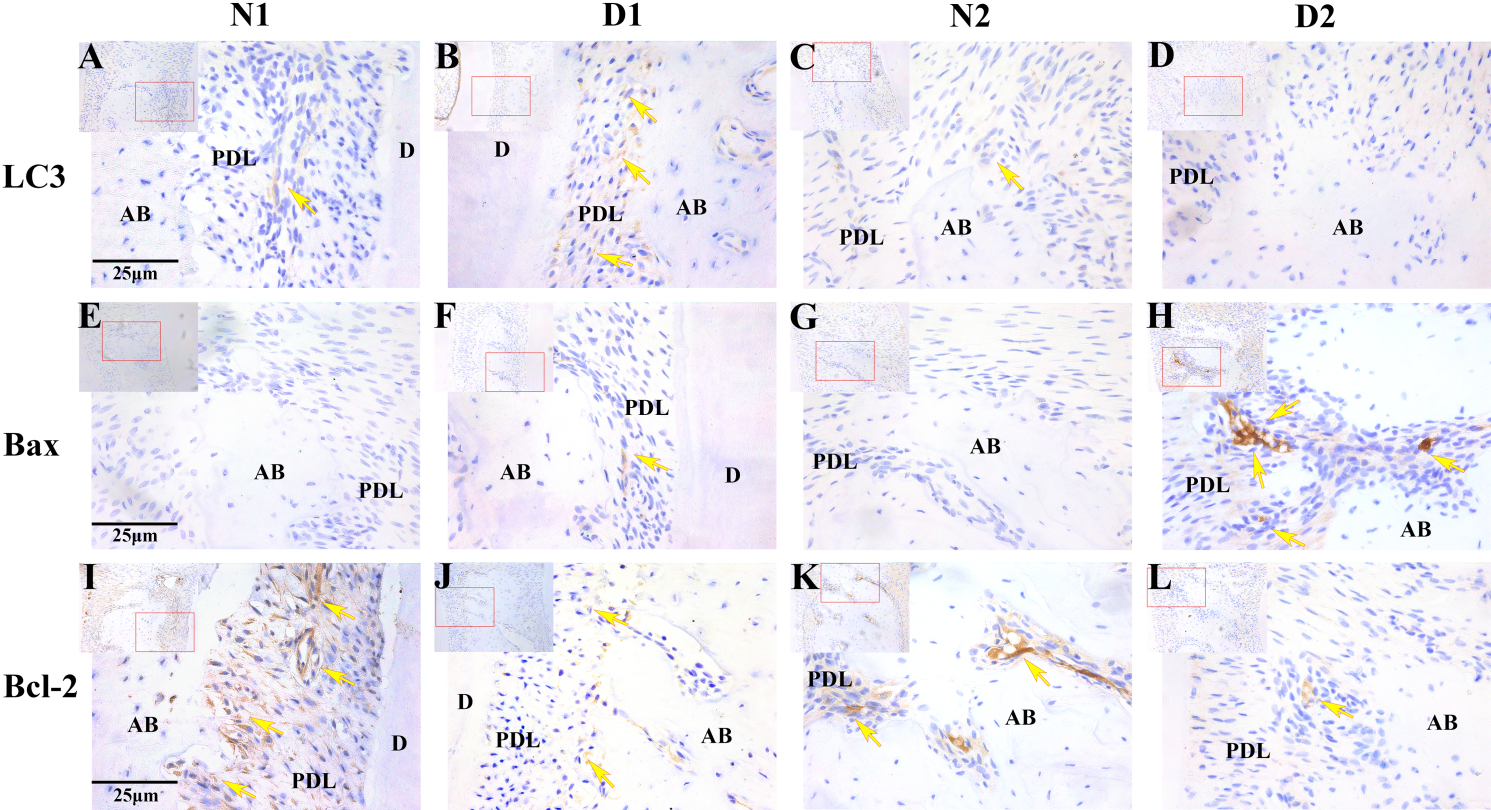
Photomicrographs of the periodontal tissue from DM rats (D1 and D2) and control groups (N1 and N2). (A–L) Photomicrographs of the periodontal tissue from DM rats (D1 and D2) and control groups (N1 and N2) showing immunoreactivity to LC3, Bax and Bcl-2. Images are shown at 40×*g* magnification. Scale bar = 25 μm. The arrow indicates high or moderate labeling in the periodontal ligament or the alveolar bone. Small picture-Upper left (shown at 20×*g* magnification).

The results of immunohistochemistry showed that LC3 was highly expressed in the D1 group and weakly expressed or not expressed in the N1, N2 and D2 groups (*P* < 0.05). The positive expression level of Bax was significantly higher in the D2 group compared to the N1, N2 and D1 group (*P* < 0.05). Bcl-2 was weakly expressed in the D1 and D2 groups and expressed more highly in the N1 and N2 groups(*P* < 0.05, Fig. 4 and Table 3).

**Table 3:** The expression of LC3, Bax and Bcl-2 in all of the groups (median ± IQR).

Variable	N1	D1	N2	D2
LC3	1 ± 0.25	3 ± 0.25*	1 ± 0*^#^	0 ± 0*^#^^Δ^
Bax	0 ± 0	1 ± 0.5*	0 ± 0	3 ± 0*^#^^Δ^
BCL2	3 ± 0	1 ± 0.25*	3 ± 0^#^	1 ± 0*^Δ^

**Notes:**

* *P* < 0.05 comparing with N1 group.

# *P* < 0.05 comparing with D1 group.

Δ *P* < 0.05 comparing with N2 group.

Each value is expressed as median ± inter-quartile range (IQR) (*n* = 6 for each group).

The degree of LC3 positive expression in the periodontal tissues of diabetic rats was negatively correlated with the degree of Bax positive expression (r = −0.915, *P* = 0.000). There was no correlation between the positive degree of LC3 and the positive degree of Bcl-2 expression (r = 0.340, *P* = 0.279). The expression of Bcl-2 was irrelevant with that of Bax (r = −0.048, *P* = 0.883).

## Discussion

DM is a common metabolic disorder with a genetic predisposition that is characterized by chronic hyperglycemia and the coexistence of various complications (Roep et al., 2021). An increasing number of studies have shown that several lesions occur in periodontal tissue in diabetes (Mealey & Ocampo, 2007; Obulareddy, Nagarakanti & Chava, 2018). In the present study, a model of DM was successfully established in rats by intraperitoneal injection of STZ. The model showed obvious inflammation and locally alveolar bone resorption through micro-CT scans and HE staining suggesting that periodontitis formation was related to diabetes. These data are consistent with most of the findings from previous studies (De Araújo et al., 2019; Fu & He, 2013; Najeeb et al., 2017).

Several studies have demonstrated that in DM, apoptosis can lead to myocardial cell destruction (Ling et al., 2013) and renal cell dysfunction (Dang et al., 2010; Xu et al., 2016a). Many studies have suggested that DM is closely related to apoptosis, oxidative stress, and inflammation in periodontal tissue (Cardoso, Reis & Manzanares-Céspedes, 2018; De Araújo et al., 2019; Fu & He, 2013; Liu et al., 2014). Apoptosis, also termed programmed cell death (PCD), is a process involved in multicellular organisms (Maiese et al., 2013). The Bcl-2 gene family plays an essential role in apoptosis and its members can have roles in pro-apoptotic and anti-apoptotic pathways. Bax is a typical representative of pro-apoptotic gene whilst Bcl-2 is a typical anti-apoptotic gene. Currently, most studies have shown that apoptosis is involved in the development of the diabetic cardiomyopathy (Guido et al., 2017), diabetic nephropathy (Han et al., 2017), diabetic retinopathy and other complications (Zhang et al., 2015). However, relatively few studies have reported apoptosis in periodontal tissue damages caused by DM.

In the current study, the expression of Bax and Bcl-2 in periodontal tissue of diabetic rats were observed by immunohistochemistry. We found that Bax and Bcl-2 were mainly expressed in the cytoplasm. The positive expression of Bax in the D2 group was significantly higher than that of the N1, N2 and D1 group. The expression of Bcl-2 was significantly lower in the N1 and N2 groups suggesting that DM promoted the occurrence of apoptosis in rat periodontal tissue by upregulating the expression of the pro-apoptotic gene, Bax, while downregulating the expression level of the anti-apoptotic gene Bcl-2.

Previous studies have shown that apoptosis and autophagy play important roles in DM (Gomes & Negrato, 2014; Gonzalez et al., 2011; Maiese et al., 2012). Recently, studies have found that the presence of chronic diabetic complications is accompanied by excessive autophagy activation in cells and oxidative stress damage may be one of the central links in the change of autophagy (Lee, Giordano & Zhang, 2012; Liu et al., 2017; Yan et al., 2012; Zhang et al., 2010).

In addition to apoptosis, autophagy is another kind of programmed cell death that plays an important role in cell growth and metabolism. In different environments or different cell types, autophagy activation may inhibit cell apoptosis, However, excessive autophagy may also cause cell death (Maiuri et al., 2007; Mariño et al., 2014; Shintani & Klionsky, 2004). Some reports suggested that autophagy may potentially inhibit apoptosis and prevent diabetic nephropathy and diabetic cardiomyopathy (Hu et al., 2015; Wei et al., 2017). However, there are relatively few reports on the role of autophagy in periodontal tissue damage caused by diabetes.

To observe the presence of autophagy in periodontal tissues, we analyzed the expression of the LC3 gene that encodes a protein involved in the process of autophagy (Bullon et al., 2012). In the present study, the level of LC3 in periodontal tissues of diabetic rats was tested by immunohistochemistry. We observed that LC3 was mainly expressed in the cytoplasm. Total levels of LC3 in the periodontal tissue of rats in the D1 group were significantly higher than that in groups N1, N2, and D2, and the expression is strongly positive. The overexpression of autophagy markers LC3 in periodontal tissues of STZ-induced diabetic rats suggested the occurrence of cell autophagy in the periodontal tissues of diabetic rats.

Studies have shown that, under certain conditions and in some experimental models, autophagy appears to play an important role in regulating cell survival rather than apoptosis (Shen et al., 2015; Wang et al., 2012). Moreover, our previous research results found that the advanced glycation end products induce autophagy of human periodontal ligament fibroblasts to protect the occurrence of apoptosis (Mei et al., 2020). However, He et al. (2013) found that autophagy and apoptosis can complement each other to control cell survival.

In the present study, the expression of Bax was weakly positive and LC3 was strongly positive in the D1 group at 4 weeks after STZ intraperitoneal injection. However, the expression of LC3 and Bcl-2 was weakly positive and Bax was strongly positive in the D2 group at 8w after STZ intraperitoneal injection. We also found that the expression of LC3 in the periodontal tissues of diabetic rats was negatively correlated with the degree of Bax expression and the inflammation of the periodontium was more significant in the group D2, which may be consistent with the results from previous studies.

The results from the present study indicated that periodontal tissues were damaged in diabetic rats. The expression of autophagy and apoptosis-related factors were detected indicating that these cellular processes might promote the damage of periodontal tissues in diabetic rats. These findings are consistent with the observations that autophagy does not continue to be beneficial for organisms (Chen et al., 2014).

In summary, the results of our present study show that overexpression of Bax and low expression of LC3 and Bcl-2 in periodontal tissues of diabetic rats may be involved in the process of periodontal tissue damage caused by diabetes. These changes may contribute to the pathogenesis of diabetic periodontal tissue injury. However, existing studies have not fully elucidated the underlying biologic mechanism through which DM may cause periodontal tissue damage. Further studies are required to elucidate the potential mechanisms between diabetes induced autophagy and apoptosis in periodontal tissue. These observations might provide a therapeutic basis for novel treatments targeting autophagy and could be another strategy for the management of periodontitis in patients with diabetes.

## Conclusions

The changes in the expression of LC3, Bax and Bcl-2 in the periodontal tissue of diabetic rats may indicate that autophagy and apoptotic are involved in the process of periodontal tissue damage and may be a mechanisms of periodontal tissue lesions.

## Supplemental Information

10.7717/peerj.11577/supp-1 Supplemental Information 1 The ARRIVE guidelines 2.0: author checklist.

10.7717/peerj.11577/supp-2 Supplemental Information 2 The raw data of Fig 2A. Alveolar bone loss was detected in M3 to M1 of each group

10.7717/peerj.11577/supp-3 Supplemental Information 3 The raw data of Fig 2B. Alveolar bone loss was detected in M3 to M1 of each group

10.7717/peerj.11577/supp-4 Supplemental Information 4 The raw data of Fig 2C. Alveolar bone loss was detected in M3 to M1 of each group

10.7717/peerj.11577/supp-5 Supplemental Information 5 The raw data of Fig 2D. Alveolar bone loss was detected in M3 to M1 of each group

10.7717/peerj.11577/supp-6 Supplemental Information 6 The raw data of 2E-CT. Alveolar bone loss detected in M3 to M1 of each group. Data represent (mean values ± SEM) (*n* = 6 for each group).

10.7717/peerj.11577/supp-7 Supplemental Information 7 The raw data of Table 3. Each value is expressed as a score of one visual field randomly selected from each section (*n* = 6 for each group)

## Additional Information and Declarations

### Data Availability

The following information was supplied regarding data availability: The raw measurements are available in the Supplementary Files. The 3D data is available at MorphoSource DOI 10.17602/M2/M360315.
